# Puberty and sex in pediatric thyroid cancer: could expression of estrogen and progesterone receptors affect prognosis?

**DOI:** 10.1530/ETJ-21-0090

**Published:** 2022-02-03

**Authors:** Julia Ramalho Amalio da Silva Breder, Paulo Alonso Garcia Alves, Mario Lucio Araújo, Barbara Pires, Priscila Valverde, Daniel Alves Bulzico, Fernanda Andrade Accioly, Rossana Corbo, Mario Vaisman, Fernanda Vaisman

**Affiliations:** 1 Endocrinology Department, Faculdade de Medicina, Universidade Federal do Rio de Janeiro, Rio de Janeiro, Brazil; 2 Pathology Department, Instituto Nacional do Cancer do Rio de Janeiro, Rio de Janeiro, Rio de Janeiro, Brazil; 3 Endocrinology Department, Instituto Nacional do Cancer do Rio de Janeiro, Rio de Janeiro, Rio de Janeiro, Brazil

**Keywords:** thyroid cancer, children, pediatric, puberty, estrogen receptor, progesterone receptor, prognosis

## Abstract

**Objective:**

A sharp increase in pediatric thyroid cancer incidence is observed during adolescence, driven mainly by girls. Differences in disease presentation across sexual maturity stages raise the question of whether sex steroids have a role in the heterogeneity. The aims of this study were to analyze the influence of puberty and sex on clinical presentation and prognosis and to evaluate the correlation between the expression of sex hormone receptors.

**Design and methods:**

Clinical records and immunohistochemical of specimens from 79 patients were analyzed. Puberty was analyzed by two criteria: end of puberty and beginning, in which the age of 10 was the cutoff.

**Results:**

Postpubertal were more frequently classified as having low-risk disease and a lower frequency of persistent disease, especially when the completion of puberty was used as the criteria. Male sex was associated with a higher risk of persistent disease at the end of the observation period. Estrogen receptor α positivity was low in the entire sample, while progesterone receptor positivity was positive in 30% of the cases. Female hormone receptor expression was not associated with sex, American Thyroid Association risk score, persistent structural disease, or pubertal status.

**Conclusion:**

Our study showed that the completion of puberty correlated best with the clinical behaviour of pediatric thyroid cancer. It was also shown that postpubertal patients have a less aggressive initial presentation and better outcomes. However, this observation could not be explained by the expression of estrogen and progesterone receptors in the primary tumors.

## Introduction

Differentiated thyroid carcinoma (DTC) is the most common endocrine tumor during childhood (1, 2). Although rare under the age of 10 years, DTC is the most common cancer in girls aged 15–19 years in the United States (3). Recent studies have reported an increased incidence of DTC in children and adolescents (1, 3). Girls are responsible for the sharply increased incidence observed during adolescence, with the girl-to-boy ratio varying from 2:1 to 5:1 in this period (3).

Previous studies have suggested some clinical differences between prepubertal and pubertal pediatric DTC. Prepubertal patients present with a greater prevalence of lymph node (4, 5) and lung metastasis (6) at diagnosis than pubertal patients. At the molecular level, Cordioli *et al.* observed a higher expression of sodium iodide symporter, pendrin, and thyroid-stimulating hormone receptor by thyroid tumor cells in adolescents compared with patients younger than 10 years (5). More recently, Sisdelli *et al.* demonstrated an association between *AGK-BRAF*fusion and distant metastasis and younger age (7).

Our group has previously shown the role of sex in pediatric DTC. In a study including 118 pediatric patients with DTC that evaluated prognostic factors for disease remission, male patients had a higher frequency of persistent disease. Other well-known prognostic factors, such as lymph node and distant metastases, were also associated with reduced disease-free survival (8).

The substantial increase in DTC incidence observed in adolescents, mainly driven by new cases in girls (4, 5, 9, 10), raises a question about the role of sex steroids in determining the heterogeneity observed in pediatric DTC. Several publications have demonstrated the presence of estrogen receptor (ER) in thyroid tumor cells in adults (11, 12, 13, 14, 15). In many studies, ERα positivity has been associated with larger primary tumor size, suggesting that estrogen may act as a growth factor in thyroid tumors (13, 14, 15). Although less frequently mentioned, the expression of progesterone receptors (PR) in DTC has also been described (13, 14, 15). Vannuchi *et al.* observed a positive correlation of tumor size and PR positivity (14), although this finding has not been replicated in other studies (13, 15).

The role of puberty, sex, and sex hormone receptors in the pathogenesis of thyroid cancer and the relationship between these variables and tumor behavior remain controversial. To date, no study has specifically evaluated the expression of ER and PR in pediatric DTC. Based on these considerations, the aims of this study were to analyze the influence of puberty and sex on the clinical presentation and prognosis of pediatric DTC and to evaluate the correlation between DTC and female sex hormone receptor expression.

## Patients and methods

### Patients

From 143 patients with pediatric DTC undergoing follow-up at the National Cancer Institute and/or Federal University of Rio de Janeiro from March 1997 to March 2019, 79 had surgical specimens to perform immunohistochemical analysis. The patients were considered to have pediatric DTC if aged ≤18 years at the time of diagnosis, according to the American Thyroid Association (ATA) guidelines (16). The surgery date was assumed as the diagnosis date. Total thyroidectomy is the standard surgery for pediatric cases at our institution, although partial thyroidectomies are exceptionally performed. Prophylactic neck dissection is not performed routinely and is only based on clinical or radiological suspicion of lymph node involvement. Most patients in our study received a therapeutic dose of radioactive iodine (RAI) after surgery, followed by suppressive therapy with levothyroxine.

The ethical review boards of both institutions involved approved the study protocol (CAAE: 66569517.8.0000.5257). Informed consent was obtained from patients and/or their parents.

### Laboratory studies

The functional sensitivity of serum thyroglobulin (Tg) assays varied throughout the study period. From 1997 to 2000, the functional sensitivity was approximately 1 ng/mL. Starting in 2001, serum Tg levels were measured by immunometric assay (Immulite, Siemens Healthineers), with a functional sensitivity of 0.2 ng/mL from 2001 to 2010 and 0.1 ng/mL after 2010.

### Immunohistochemical analysis

Immunohistochemical analysis was performed on the primary tumor specimens of all 79 patients. All histopathological diagnoses were reviewed by an expert pathologist. Formalin-fixed paraffin-embedded tissue sections (3 μm) were dewaxed in xylene and rehydrated in graded ethanol solutions. Antigen retrieval was performed in Trilogy buffer (Cell Marque, Merck) at 98°C using the steam process for 30 min. Endogenous peroxidase and protein blockade were performed by Novolink Max Polymer Detection (Leica Microsystems). Subsequently, the sections were incubated overnight with specific primary antibodies (Dako ERα: clone 1D5, dilution 1:2000; Dako, PR: clone 636, dilution 1:2000). The reaction was detected with a Novolink Max polymer detection system (Novocastra Laboratories Ltd, Leica Microsystem) following the manufacturer’s instructions and using diaminobenzidine as a chromogen. The sections were counterstained with hematoxylin. Ductal breast carcinoma was used as a positive and negative control for the reactions. The primary antibody was omitted to provide negative controls.

Immunohistochemical staining was analyzed under a high-power field (×40) using a standard light microscope to observe nuclear marking. The results from the immunohistochemical analysis were classified using the Allred score. The percentages of ERα- and PR-stained nuclear cells were categorized as zero if there was no staining, one if stained nuclei were less than 1% of all nuclei, two if 1–10%, three if 11–33%, four if 34–66%, and five if > 66% stained nuclei were observed. Staining intensity was scored as 0 if is there was no staining, 1 if staining was weak, 2 if it was moderate, or 3 when strong staining was observed. The total score was calculated by adding the percentage and intensity scores, with the final result ranging from 0 to 8. For statistical purposes, ERα and PR status were deemed positive when the total score was ≥2.

### Evaluation of outcomes

Clinical and pathological features and data from treatment, course, and outcome were collected from medical charts. Extrathyroidal extension was based on American Joint Committee on Cancer (AJCC) 7th edition. Additional therapy was defined as more than one RAI treatment or extra surgery.

To better understand the importance of puberty in DTC, we stratified prepubertal and pubertal patients. Since Tanner staging was not available in all patients, we proposed two criteria. In pubertal criterion 1, patients were stratified into prepubertal and pubertal groups according to the age of ten years. In pubertal criterion 2, puberty was defined as the occurrence of menarche in girls and the age of 16 years in boys. This cutoff was based on a Brazilian study (17) in which boys reached the G4 Tanner stage by age 16 years, in parallel to the final phase of puberty in girls represented by menarche.

Regarding outcomes, patients were considered to have no evident disease (NED) at the final follow-up if stimulated serum Tg was <2 ng/mL or suppressed serum Tg was <1 ng/mL in the absence of structural evidence of disease. Patients with suppressed serum Tg >1 ng/mL and no evidence of structural disease were deemed to have biochemical persistence. Patients were classified as having structural disease if they had biopsy-proven cervical disease or clinical suspicion associated with cross-sectional or functional imaging suggestive of distant involvement. As structural disease is associated with more aggressive treatment, we dichotomized the sample among those with structural disease versus those with an absence of structural disease (NED plus biochemical persistence) to better study the risk factor for this spectrum of disease. The final status was determined by data from the last medical visit.

### Statistical analysis

The descriptive analysis was expressed by measures of central tendency and dispersion suitable for numerical data and by frequency and percentage for categorical data.

The inferential analysis was composed to compare numerical data by ANOVA Kruskal–Wallis between outcomes with three subgroups and the Mann–Whitney test between outcomes with two subgroups (18). The Dunn multiple comparison test was applied to identify which subgroups differed significantly from each other (19) Categorical data were compared using the chi-square test (χ^2^) or Fisher’s exact test.

The Kaplan–Meier method was applied to estimate disease-free survival related to puberty and sex and compared by log-rank statistics.

The normality of the distribution of the data was assessed by the Shapiro–Wilk test and graphical analysis of histograms. Differences were considered statistically significant when *P* values were equal to or less than 0.05. The analysis was performed using SPSS version 26.

## Results

### Clinical and histopathological features

Demographic, clinical characteristics, and initial management data are summarized in Table 1. The median age at diagnosis was 14, and the youngest patient was 4 years old. Most patients were girls (64.6%). Most of the sample was postpubertal at diagnosis, including 83.5% if pubertal criteria 1 (cut point 10 years) was adopted and 62% if criteria 2 (menarche for girls and 16 years in boys) was used. The median age at menarche was 12 years. More than 90% of the sample underwent total thyroidectomy and received RAI therapy.

**Table 1:** General characteristics.

	*n*= 79	%
	Mean ± s.d.	
	Median (min–max; IQR)	
Age at diagnosis (years)	13.9 ± 3.7	–
	14 (4–18; 12–17)	
Gender (female:male)	51:28	64.6:35.4
Menarche age (years), *n* = 51	12 ± 1.4	–
	12 (8–15; 11–13)	
Diagnosis after menarche		
Yes	41	80.4
No	10	19.6
Puberty by criteria 1		
Yes	66	83.5
No	13	16.5
Puberty by criteria 2		
Yes	49	62
No	30	38
Total thyroidectomy		
Yes	76	96.4
No	3	3.8
Histopathology		
Papillary thyroid carcinoma	77	97.5
Hurthle thyroid carcinoma	2	2.5
Tumor size (cm), *n* = 76	2.8 ± 1.4	–
	2.65 (0.5–7; 2.0–3.4)	
Extra-thyroidal extension, *n* = 76		
Yes	50	64.1
No	28	35.9
Multifocality, *n* = 77		
Yes	39	50.6
No	38	49.4
Lymph node metastasis, *n* = 75		
Yes	55	73.3
No	20	26.7
Number of positive lymph nodes	9.4 ± 9	–
	7 (0–39; 3–14)	
Distant metastasis		
Yes	19	24.1
No	60	75.9
Hypoparathyroidism, *n* = 73		
Yes	36	49.3
No	37	50.7
Estrogen receptor		
Yes	11	13.9
No	68	86.1
Progesterone receptor		
Yes	24	30.4
No	55	69.6
TNM		
Stage 1	19	24.1
Stage 2	60	75.9
ATA Ped risk		
High	34	43
Intermediate	18	22.8
Low	27	34.2
RAI		
Yes	75	94.9
No	4	5.1
Cumulative RAI activity (mCi)	237 ± 189	–
	150 (0–1000)	
Additional therapy		
Yes	23	29.1
No	46	70.9
Follow-up (years)	7.9 ± 5	
	6.4 (0.8–20.7; 3.7–11.2)	
Time to NED (years)	4 ± 4.5	
	2 (0.30–20.1; 1.1–5.5)	
Final status		
NED	52	65.8
Biochemical incomplete	12	15.2
Structural persistence	15	19
Disease-related death	0	0

SD, standard deviation; IQR, interquartile range (Q1–Q3); Puberty by criteria 1, cutoff 10 years; Puberty by criteria 2, menarche for girls and 16 years for boys; Extra-thyroidal extension, considering 7th ed AJCC; TNM, TNM Classification of Malignant Tumours 8th ed; ATA Ped risk, American Thyroid Association Pediatric Thyroid Cancer Risk Levels for recurrent/persistent disease; RAI, radioactive iodine; Additional therapy, more than one RAI or new surgery; NED, no evidence of disease; Final status, situation at the last medical visit.

Of the 79 cases, papillary thyroid carcinoma comprised 77 cases, and the 2 remaining cases were Hurthle thyroid carcinoma. Regarding the initial presentation, lymph node involvement was found in 73.3% of the patients, and distant metastasis was found in 24.1% of them. All distant metastases occurred in the lungs. Based on the pediatric risk stratification by the ATA (16), 43, 22.8, and 34.2% of the patients were classified as having high-, intermediate-, and low risk for persistent disease, respectively. After a median follow-up of 6.4 years (0.8–20.7 years), 52 (65.8%) patients were classified as having NED, 12 (15.2%) as presenting biochemical persistence, and 15 (19%) as having a persistent structural disease. Twenty-three patients were submitted to additional therapy.

### Outcome

Younger age was statistically associated with high-risk ATA compared to low-risk ATA (median age for high risk 13 years vs 15 for intermediate vs 16 for low risk, *P*  = 0.005). There was no association of pubertal status by pubertal criteria 1 (10 years cutoff) to ATA risk classification or final status. Otherwise, considering pubertal criterion 2 (Table 2), lymph node and distant metastases were more common at diagnosis in the pre- or peripubertal group, p values of 0.016 and 0.04, respectively. Being pre- or peripubertal by criteria 2 was also statistically associated with high-risk ATA classification for persistent/recurrent disease (*P*  = 0.003). Regarding treatment, there were no differences in cumulative RAI activity, additional therapy, and early excellent treatment response (6–12 months) related to puberty status. Patients classified as ATA high risk were more frequently submitted to additional therapy.

**Table 2:** Clinical presentation and prognosis vs puberty status at diagnosis (criteria 2). Continuous variables were expressed as median and minimum and maximum range and compared using Mann–Whitney test. Categorical variables were expressed as frequency and compared using χ^2^ or Fisher’s exact test.

	Pre and peri puberty (*n* = 30)	Post puberty (*n* = 49)	*P* -value
Tumor size (cm)	2.88 (0.7–6.4)	2.69 (0.8–7.0)	0.23
ETE	65%	63%	0.84
Multifocality	55.2%	48%	0.53
Lymph node metastasis	89.3%	63.8%	**0.016**
Distant metastasis	36.7%	16.3%	**0.04**
ATA high-risk score	66.7%	28.6%	**0.003**
Excellent response in 6–12months	37.9%	34.2%	0.8
Cumulative RAI activity (mCi)	200 (0–800)	150 (0–1000)	0.73
Additional therapy	44.4%	25.8%	0.11
Final status			
NED	58%	69.5%	0.46
Biochemical incomplete	12%	18.3%	0.06
Structural persistence	30%	12.2%	**0.05**

ETE, extrathyroidal extension considering 7th ed AJCC; ATA, American Thyroid Association; RAI, radioactive iodine; Additional therapy, more than one RAI or new surgery; Final status, situation at the last medical visit; NED, no evidence of disease.

Bold indicates statistical significance, *P* < 0.05.

Focusing on structural disease risk factors (Table 3), boys had a greater risk of structural persistence (*P*  = 0.027) as well as being pre- or peripubertal at diagnosis by criteria 2 (*P*  = 0.05). Nodal and distant metastases, local invasion, cumulative RAI activity, and additional therapy were also positively associated with structural persistence. The number of positive lymph nodes at presentation was positively associated with structural disease at the final follow-up (median 15.5 positive nodes (interquartile range (IQR) 6.8–19.3 in the persistent group vs six positive nodes, (IQR 2–10) for no persistent *P*  = 0.002).

**Table 3:** Risk factors for persistent structural disease. Data were expressed as frequency (*n*) and percentage (%).

Variable	Structural persistence		No structural persistence		*P* value^a^
	*n*	%	*n*	%	
Gender					
Male	9	60.0	19	29.7	**0.027**
Female	6	40.0	45	70.3	
Puberty criteria 1					
Yes	12	80.0	54	84.4	0.47
No	3	20.0	10	15.6	
Puberty criteria 2					
Yes	6	40.0	43	67.2	**0.050**
No	9	60.0	21	32.8	
Lymph node metastasis					
Yes	15	100.0	40	66.7	**0.005**
No	0	0.0	20	33.3	
Extrathyroidal extension					
Yes	14	93.3	36	57.1	**0.008**
No	1	6.7	27	42.9	
Multifocality					
Yes	11	73.3	28	45.2	**0.050**
No	4	26.7	34	54.8	
Distant metastasis					
Yes	12	80.0	7	10.9	<0.0001
No	3	20.0	57	89.1	
TNM					
Stage 1	3	20.0	57	89.1	<0.0001
Stage 2	12	80.0	7	10.9	
Additional therapy					
Yes	11	73.3	12	18.8	<0.0001
No	4	26.7	52	81.3	
Estrogen receptor					
Yes	2	13.3	9	14.1	0.65
No	13	86.7	55	85.9	
Progesterone receptor					
Yes	6	40.0	18	28.1	0.27
No	9	60.0	46	71.9	

^a^χ^2^ or Fisher’s exact test.

No structural persistence, no evidence of disease plus biochemical persistence; Puberty criteria 1, cutoff 10 years; Puberty criteria 2, menarche for girls and 16 years for boys; Extra-thyroidal extension, 7th ed AJCC; TNM, 8th ed AJCC classification system; Additional therapy, more than one radioactive iodine therapy or new surgery.

Bold indicates statistical significance.

**Figure 1:**
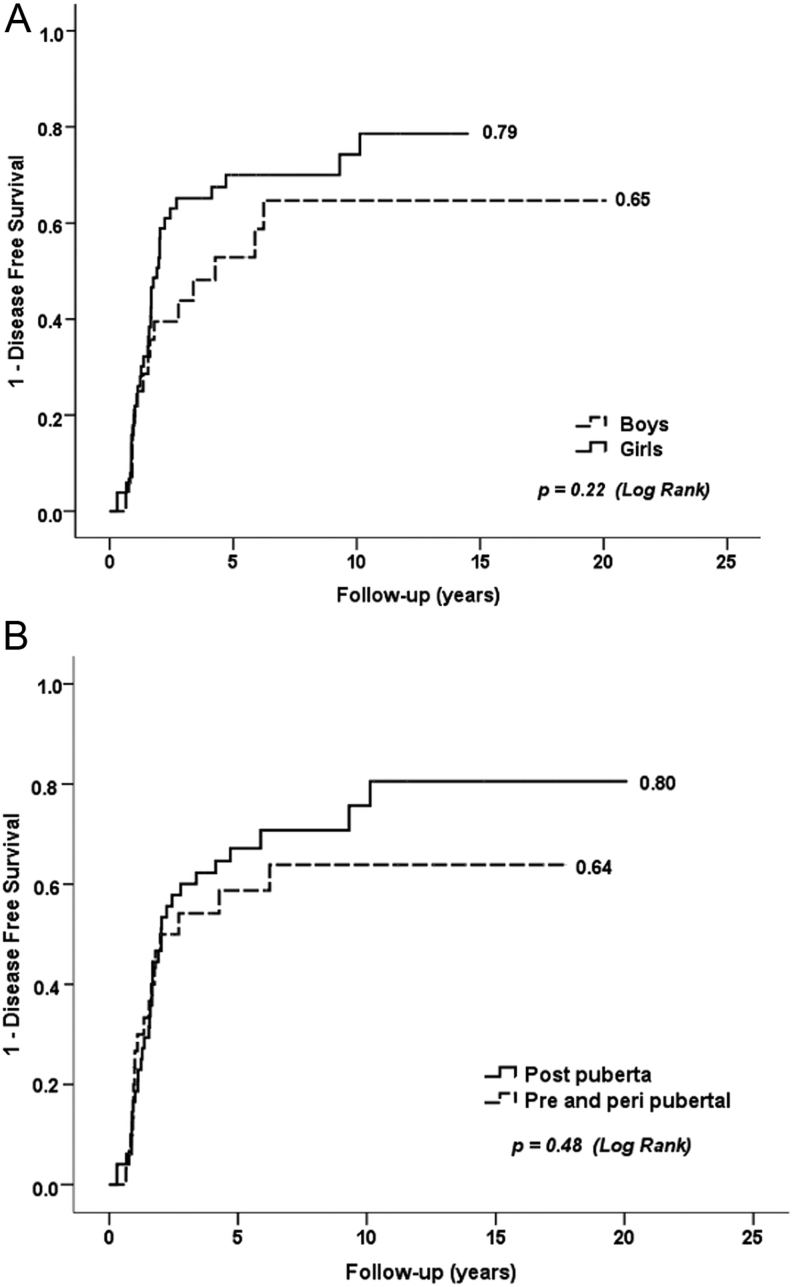
(A) Sex vs disease-free survival. (B) Puberty completed at diagnosis vs disease-free survival.

There was no difference in disease-free survival related to sex or pubertal status, as shown in Kaplan–Meier curves (Fig. 1, Kaplan–Meier curve disease-free survival by sex (A) and puberty by criteria 2 (B)). The current survival rate of our cohort is 100%.

### Immunohistochemical expression of ERα and PR

**Figure 2:**
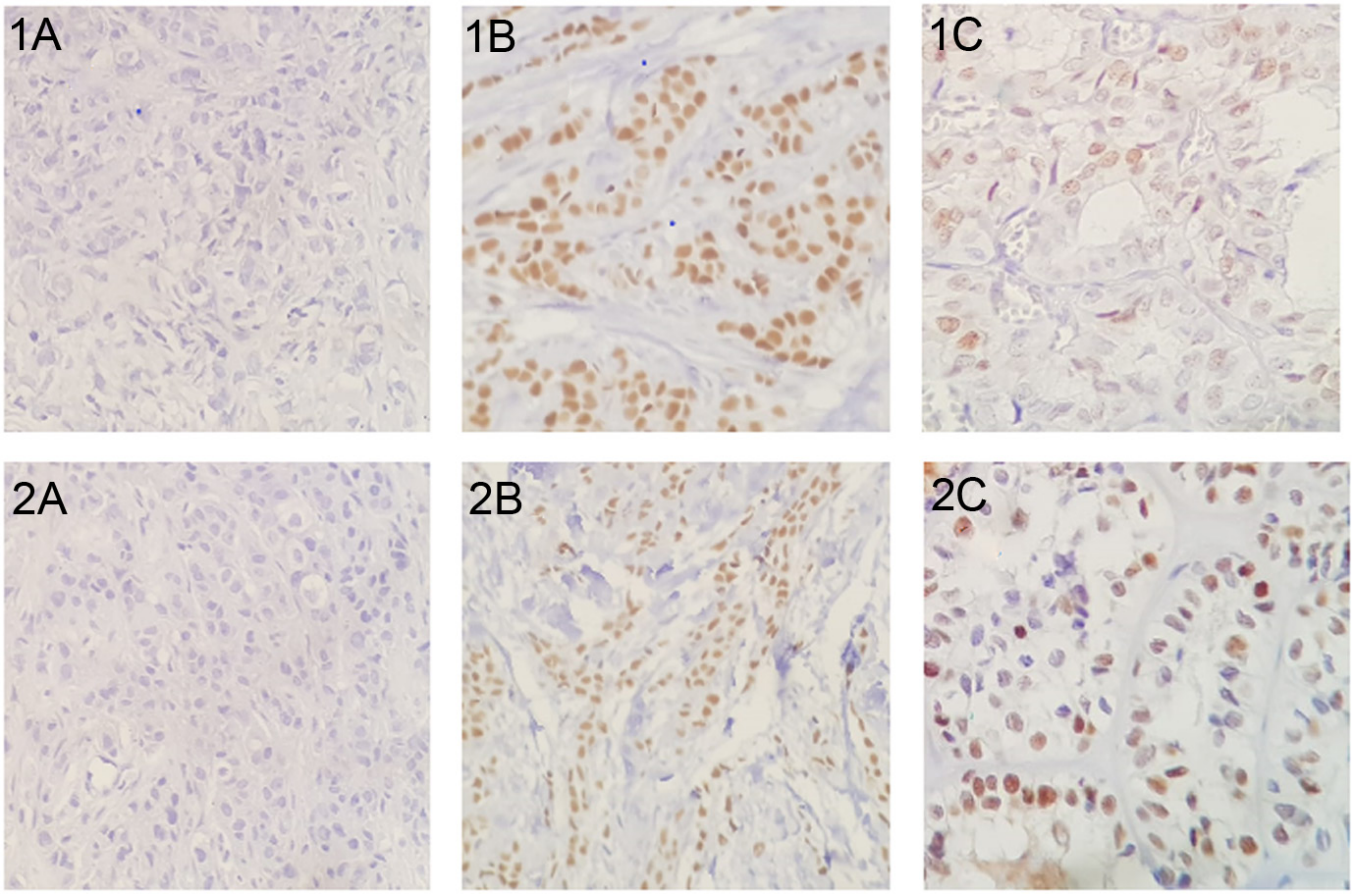
Immunohistochemistry for ER and PR. (1A) RE negative control; (1B) RE positive control; (1C) RE moderate intensity; (2A) RP negative control; (2B) RP positive control; (2C) RP strong intensity.

From all 79 studied patients, 13.9 and 30.4% of cases were positive for ERα and PR staining, respectively (Table 1). No difference in ERα or PR expression was observed between sexes, pubertal stages, age (Tables 4 and  5), structural vs no structural persistence (Table 3), or ATA risk classification. ERα-positive cases were mostly focal (<1% of the cells per field) and with weak intensity (Fig. 2). When Allred score was analyzed, there was also no difference among the groups, neither regarding intensity nor number of marked cells. In PR-positive tumors, nuclear staining intensity varied from weak to strong (Fig. 2). Regarding the percentage of marked cells, the distribution of the positive cells varied from less than 1% to >66% of the cells. Interestingly, the two cases of Hürthle cell carcinoma in the study had PR expression with strong nuclear staining on a large extension of the tumor, that is, 34–66% and >66% of the cells.

**Table 4:** Estrogen receptor expression and patient characteristics. Data were expressed as frequency (*n*) and percentage (%) and compared using Fisher’s exact test. Age (in years) was expressed as median and interquartile range (Q1–Q3) and compared using the Mann–Whitney test.

Variable	ER positive		ER negative		*P* value^a^
	*n*	%	*n*	%	
Gender					
Male	4	36.4	24	35.3	0.59
Female	7	63.6	44	64.7	
Puberty criteria 1					
Yes	11	100	55	80.9	0.12
No	0	0	13	19.1	
Puberty criteria 2					
Yes	8	72.7	41	60.3	0.33
No	3	27.3	27	39.7	
Age (years)					
Median (Q1–Q3)	16 (14.5–17)		14 (12–17)		0.086

ER, estrogen receptor; Puberty criteria 1, cutoff 10 years; Puberty criteria 2, menarche for girls and 16 years for boy.

^a^χ^2^ or Fisher’s exact test.

**Table 5:** Progesterone receptor expression and patient characteristics. Data were expressed as frequency (*n*) and percentage (%) and compared using Fisher’s exact test. Age (in years) was expressed as median and interquartile range (Q1–Q3) and compared using the Mann–Whitney test.

Variable	PR positive		PR negative		*P* value^a^
	*n*	%	*n*	%	
Gender					
Male	11	45.8	17	30.9	0.20
Female	13	54.2	38	69.1	
Puberty criteria 1					
Yes	21	87.5	45	81.8	0.39
No	3	12.5	10	18.2	
Puberty criteria 2					
Yes	13	54.2	36	65.5	0.34
No	11	45.8	19	34.5	
Age (years)					
Median (Q1–Q3)	15 (12–17)		14 (12–17)		0.54

ER, estrogen receptor; Puberty criteria 1, cutoff 10 years; Puberty criteria 2, menarche for girls and 16 years for boys.

^a^χ^2^ or Fisher’s exact test.

## Discussion

In the present study, pre- or peripubertal patients had higher ATA recurrent/persistent risk scores and a greater frequency of structural persistent disease at the final follow-up. Male sex was also a risk factor for structurally persistent disease. Although boys and pre- or peripubertal individuals had a higher prevalence of persistent structural disease, no difference in disease-free survival was observed between sexes and pubertal status.

Other authors have also shown increased aggressiveness at presentation in patients diagnosed with DTC before puberty (4, 5, 6, 20, 21, 22, 23, 24). In a sample of 27 pediatric patients, including 10 prepubertal patients, Lazar et al. found nodal metastasis at diagnosis in all prepubertal patients and distant metastasis in 70% of them (4). More recently, Galuppini et al. showed a higher prevalence of ETE and nodal and distant metastases at diagnosis among the youngest patients in their study (23). Despite the findings of these studies, some authors found no association between puberty and clinical and pathological features in DTC (10, 25). In agreement with previous studies (5, 21, 22, 26, 27), structural disease at the end of follow-up was more prevalent in prepubertal patients, despite no difference in the long-term disease-free survival rate.

The conflicting data regarding puberty and DTC could be related to a great diversity in criteria used to classify puberty. The most used criterion is stratification by age, although age cutoff values vary (5, 10, 19, 21, 22, 23, 25, 26, 27). Only a few authors have classified pubertal groups according to Tanner stage (4, 20). In our study, Tanner stage was not available for all patients, so we chose to classify patients’ pubertal status by the occurrence of menarche in girls to utilize a clinical marker of puberty. Furthermore, previous studies have shown that Tanner stages may not accurately assess pubertal development when compared to a hormone-derived pubertal assessment method (28). Menarche is usually registered in medical charts and, otherwise, is easily memorable. In contrast to girls, boys do not have an easy-to-recall clinical marker of puberty, so we needed to stratify by age. As previously reported, we used Brazilian population-based studies in healthy children to establish the best age for boys. That study showed that the mean age at which testicles reached adult volume was 15.8 years (ranging from 15.3 to 16.3 years). We also stratified pubertal status by age of ten (pubertal criteria 1), a usual cutoff point. Interestingly, pubertal status by criteria 1 was not associated with a difference in the final follow-up, while being postpubertal by criteria 2 (menarche for girls and 16 years for boys) at diagnosis was associated with a lower frequency of persistent structural disease (*P*  = 0.05). Since 65% of the sample was composed of girls, most of the criteria 2 pubertal status were determined by clinical parameters. As puberty is a dynamic phenomenon, it does not seem reasonable to have a cutoff based on age to determine whether one is pre or post-pubertal. Based on our findings, we believe that the pubertal criterion by age is definitively inappropriate for retrospective studies. In the absence of Tanner stage information, we believe that clinical criteria are better at identifying pubertal status than chronological criteria.

From a genetic perspective, the more aggressive presentation of pediatric DTC in prepubertal patients may be explained by recent findings of different mutation profiles in children compared with adults. In younger children, fusions seem to be more frequent, while classic mutations such as BRAF V600E and TERT are rare (23, 29). In a study of 80 pediatric patients, Sisdelli *et al.* found a higher prevalence of *AGK-BRAF* fusion in children younger than 13 years. The occurrence of *AGK-BRAF* fusion correlated positively with lung metastasis (7). In another recent publication, Pekova *et al.* found fusion events in 56% of their pediatric sample, with *RET* fusion being the most common type (29). *RET* fusion has been associated with more frequent lymph node involvement, distant metastasis, and prepubertal age (24). In this cohort, *RET* fusion-positive patients had no history of previous radiation.

Regarding sex differences, our findings corroborate those of previous studies (4, 10, 21, 22, 26) that have shown a predominance of DTC among girls and mainly after puberty. Although girls are more frequently affected by the disease, the current findings confirmed those of our previous study (8), which demonstrated that the male sex is a risk factor for persistent structural disease (*P*  = 0.027). In addition to male sex, positive lymph nodes (*P*  = 0.005), the presence of ETE (*P*  = 0.008), multifocality (*P*  = 0.05), and distant metastasis (*P*  < 0.0001) were significantly associated with persistent structural disease.

To date, no study has investigated the presence of ERα and PR in pediatric DTC. In our sample, the positivity of ERα (13.9%) and PR (30.4%) had a lower rate than expected. In adults, ERα and PR positivity rates range from 20 to 100% and from 40 to 75%, respectively (12, 13, 14, 15, 30). As in adult studies (13, 14, 15, 30), no difference between sexes was observed. Although studies on breast cancer have reported a more aggressive profile and less frequent expression of sex hormone receptors in adolescents’ tumors (31), in the present study, we were unable to determine if the low rate of expression of sex hormone receptors in pediatric DTC is a phenomenon associated with childhood.

Based on our findings, the presence of ERα and PR was not implicated in differences observed between sex and pubertal status in pediatric DTC. We did not evaluate the presence of testosterone receptors, which could underlie the sex difference findings. Even though less studied, the presence of androgen receptors in thyroid cancer has been described as a possible mechanism for sex distinction (32, 33, 34). In a study with transgenic mice that mimicked human follicular thyroid cancer (FTC), sham-orchiectomized males had larger tumors than orchiectomized animals. When the genetic profile was analyzed, females and castrated males had a similar genetic expression, which was different from that of noncastrated males. Most of the differentially expressed genes contain testosterone receptor binding sites and are related to tumor suppressor and immune-regulatory activity. To further evaluate the influence of testosterone, testosterone was replaced in a group of castrated mice, and the reconstitution of testosterone in castrated mice reversed the gene expression profile to that of sham-castrated males (33). In another study, Stanley described sex differences in testosterone levels and androgen receptor expression in human thyroid carcinoma, claiming a specific sex modulation of androgen in thyroid tumors (34).

This study has some limitations. Since the Tanner stage was not available in all cases, menarche was considered the mark of puberty in girls, while chronological criteria were used for this purpose in boys. Another limitation of our study was that surgical specimens were not prepared for estrogen and progesterone staining, which may have also influenced our low positivity rate. In favor of our study, most adult studies on ER and PR in DTC also utilized paraffin-embedded tissue from previous surgery with no specific preparation for ER and PR immunohistochemistry. Also, the antibody used was not able to detect ERβ, which, besides being less frequent could also be associated with prognosis.

In conclusion, our study showed that pre- and peripubertal patients had higher ATA persistent/recurrent risk scores and an increased frequency of persistent structural disease at the final follow-up. As Tanner staging is frequently not available and cannot always be accurate to assess pubertal development when compared to the hormone-derived pubertal assessment method, we propose that the end of puberty could be used as a good prognostic marker. Confirming a previous publication from our group (8), male sex was a risk factor for persistent structural disease, although this could not be explained by the expression of ERα and PR in the primary tumors. Future studies are needed to evaluate a possible modulation of estrogen and progesterone in tumor suppressor genes to explain the higher prevalence of female cases in the postpubertal period and the greater aggressiveness in males. A better understanding of the role of puberty and sex in DTC could influence the elaboration of specific therapeutic recommendations. As with other types of cancer, knowledge of ER and PR expression and the influence of their expression on pediatric DTC may reveal new therapeutic targets.

## Declaration of interest

The authors declare that there is no conflict of interest that could be perceived as prejudicing the impartiality of the research reported.

## Funding

This work was funded with Vaisman F grant from FAPERJ number E_10/2016E.
